# A study on the equality and benefit of China’s national health care system

**DOI:** 10.1186/s12939-017-0653-4

**Published:** 2017-08-29

**Authors:** Shaoguo Zhai, Pei Wang, Quanfang Dong, Xing Ren, Jiaoli Cai, Peter C. Coyte

**Affiliations:** 1 School of Public Administration, Northwest University, 1 Xuefu Road, Chang’an District, Xi’an, Shaanxi, 710127 China; 2 School of Politics and Administration, Xianyang Normal University, Wenlin Road, Xianyang, Shaanxi 712000 China; 3 School of Public Policy and Administration, Xi’an Jiaotong University, 28 West Xian ning Road, Beilin District, Xi’an, Shaanxi, 710049 China; 4 School of Economics, Wuhan University of Technology, 122 Luoshi Road, Wuhan, Hubei 430070 China; 5 Institute of Health Policy, Management and Evaluation, University of Toronto, Health Sciences Building, 155 College Street, Suite 425, Toronto, ON M5T3M6 Canada

**Keywords:** Health care, Equality, Benefit

## Abstract

**Background:**

This study is designed to evaluate whether the benefit which the residents received from the national health care system is equal in China. The perceived equality and benefit are used to measure the personal status of health care system, health status. This study examines variations in perceived equality and benefit of the national health care system between urban and rural residents from five cities of China and assessed their determinants.

**Methods:**

One thousand one hundred ninty eight residents were selected from a random survey among five nationally representative cities. The research characterizes perceptions into four population groupings based on a binary assessment of survey scores: high equality & high benefit; low equality & low benefit; high equality & low benefit; and low equality & high benefit.

**Results:**

The distribution of the four groups above is 30.4%, 43.0%, 4.6% and 22.0%, respectively. Meanwhile, the type of health insurance, educational background, occupation, geographic regions, changes in health status and other factors have significant impacts on perceived equality and benefit derived from the health care system.

**Conclusion:**

The findings demonstrate wide variations in perceptions of equality and benefit between urban and rural residents and across population characteristics, leading to a perceived lack of fairness in benefits and accessibility. Opportunities exist for policy interventions that are targeted to eliminate perceived differences and promote greater equality in access to health care.

## Introduction

The Chinese National Health care System has been being reformed substantially in recent year from the aspects of its organization and financial resources. Access to health services and health insurance, for instance, is currently stratified demographically with separate streams for rural, urban working, and urban non-working residents. However, trade-offs between ‘access, quality and cost’ are triggering significant challenges, with inequalities between urban and rural populations chief among them [1–3]. The consequence, for some, is ‘health poverty’ which is often attributed to the inability to access affordable basic health care services [4].

Although it is impossible to completely eliminate health inequities via policy changes, government has obligations and responsibilities to try their best [5, 6]. In China, however, further research is needed to identify possible regional and social-economic differences concerning the access to health and health quality before recommended changes of suitable policy [7–9].

The purpose of this study is twofold: first, to explore perceptions among urban and rural residents concerning the equality and benefit of China’s national health care system; and, second, to analyze underlying factors that influence those perceptions. ‘Perceived equality’ is defined as the degree to which respondents reported a sense of fairness within the national health system; and ‘perceived benefit’ is defined as the degree to which respondents reported satisfaction with various aspects of the national health care system.

In this research, we present survey resulting from a sample of Chinese urban and rural residents from five economically and geographically representative regions. We also classify the whole sample by type of health insurance, education, occupation and regions to identify factors that may explain variations in perceived equality and benefit within China’s health care system.

## Background

China’s current health care system is designed to serve three distinct groups of care recipients: first, basic health insurance for urban workers (with insurance premiums paid by these workers and their employers); second, basic health insurance for non-working urban residents (with insurance premiums paid primarily by those residents with small government insurance premium subsidies); and finally, rural cooperative health insurance for rural residents (with insurance premiums paid by those residents, complemented by a collective allowance as well as a subsidy from central and local government). Variations concerning access and quality of health services between urban and rural residents have attracted considerable attention. Fang et al. [10] pointed out that urban residents appear to fare worse than rural residents in terms of overall health status and health care utilization in China by identifying the causes of urban-rural health disparities. Liu et al. [11] described patterns in physician and hospital utilization among rural and urban populations in China and explored factors associated with differences, expounding that the medical conditions such as facilities and techniques of practitioners are very different between rural and urban. Magadi et al. [12] used Demographic and Health Survey data from 23 countries in Sub-Saharan Africa to show that the care of urban poor is worse than that of the urban non-poor, and the study suggested that the urban bias in the allocation of health services in Africa does not benefit the urban-poor as much as the non-poor. Smith [1] analyzed China’s modernization, health service system and reported growing inequality between urban and rural areas. Liu et al. [2] analyzed inequality in access to health care between urban and rural areas caused by various health reforms in China’s health system. Zhang et al. [3] stated that the foundation of education and health care has been altered in the era of economic reform, so that social inequalities have increased dramatically among provinces, cities, and urban and rural areas. Prahlad et al. [13] conducted a survey of public health facilities satisfaction and concluded that measuring patient satisfaction were more satisfied with the basic amenities at higher health facilities compared to lower level facilities. It was also observed that the patients were more satisfied with the behavior of doctors and staff at lower health facilities compared to higher level facilities. Fonseca et al. [14] argued that customer satisfaction should be measured through multiple indicators, as a latent variable and considered the latent segment models (LSM) approach to assess customer service satisfaction for public health care. Lech [15] surmised that level of satisfaction from the services provided by health care facilities did not correlate with gender or age of the respondents. In opposite, the (higher) level of education and place of living (in a big city) have negatively correlated with patients satisfaction. Devi et al. [16] examined the predictors and level of patients’ satisfaction with health care quality across the five regional hospitals of Mauritius with multiple regression analysis, which offered a reference to methods in our research. Moises [17] examined user perceptions of health care delivery in selected rural and urban areas of three Central American countries. 351 residents from rural and urban communities represented different genders, ages, occupations, health, and socio-economic status. Participants’ main preoccupations focused on prompt access to trusted physicians, effective and inexpensive medication, and quality attention in public hospitals hoping for improved medical services. The rural poor, especially indigenous people, voice basic needs with little regard for quality.

Previous studies about access and quality of health services between urban and rural residents were limited to examining health insurance and health care in certain areas in China. A growing number of studies addressed geographic variation in access to health care services and outcomes. Considering that there exists little empirical evidence on the disparity of different groups of populations by distinguishing their degree of equality and benefit of China’s national health care system, we conduct this unique study to fill this knowledge gap in China. The study uses a wider survey among typical areas to describe patterns of populations which were affected by the rural and urban health care system deeply, and to identify factors associated with any observed differences. The study provides evidence to health care policy makers on designing and operating this national system to make people in different areas achieve more benefit and more equal access to the health care system.

With the basic aim of maintaining and improving the level of national health status, medical insurance system should be guided by and centered on health, so it is necessary to do the literature review about health and its influencing factors. It is generally acknowledged by research that the definition and measurement of health are very complex because it is a subjective concept with objective features, which are influenced by multi-factors, and there still exists health inequality objectively. Wang [18] summarized that health is the perfect condition in physical, mental and social adaptation. Indexes such as life expectancy, self-rated health scores, death rate, and morbidity are often used to measure the effects of medical intervention and the improvement of health conditions. Self-evaluation on equality, benefit based on self-rated health is most easily obtained from individual samples, so it is widely used in the research Grossman [19]. Yang and Liu [20] hatched the idea that health is the integrated result of a variety of factors mainly including genetic characteristics, such as gene, gender, race and age; environmental factors such as natural environment (including water, soil, climate, air, etc.); social environment (including income, social status, modernization, urbanization, etc.); medical insurance factors and lifestyle factors. According to their research, among these factors, genetic factors account for 15%, while environmental factors and medical insurance factors account for 17% and 8% respectively, with behavior and lifestyle of a person such as living habit, health behavior, spirit, health awareness, taking up to 60%. Based on the data, scholars have conducted related research on the influencing factors of health from age, gender, race, income, education and behavior Rahkonen et al. [21]. Overall, recent research on the factors of health emphasizes on environment, behavior, and genetics, while few research has been conducted on medical insurance which mainly focuses on the effect of medical technology and services on health. The related health improving mechanism has not been studied systematically. The goal of health care equality after the year of 2000 makes health care insurance become a hot issue again. The related researches mainly focused on the unfair relations between health and socioeconomic status such as income, location, gender and assets Chen et al. [22]. Therefore, in order to promote equality of health care, it is necessary to improve the financing and payment mechanism.

## Methods

A survey, designed to assess levels of perceived health equality and benefit, was administered between July and August of 2012. This survey is a cross sectional one based on individuals in household and we design our final statistical report according to STROBE guidelines’ list. Respondents comprised urban and rural residents from five cities of China including Zhenjiang, Dongguan, Chengdu, Shenmu, and Yinchuan. Although five cities from eastern and western china, they are typical representative samples of China and were chosen based on their geographical location and degree of economic development by the Delphi Method, which is the expert consultation for two rounds of inquiry by gathering experts from domains of general medicine, clinical medicine, health education and health economics to screen and select these samples.

### Procedure

We utilize data from the survey sponsored by the National Social Science Fund. A multistage sampling method was adopt in 2012 to achieve representation of the samples. The sampling classified into 3 stages. In the first stage, 31 provinces in China were divided into four parts geographically, namely eastern, western, northern and southern regions, which are clustered by the government administrative geographic system (ie, city, county, town, and village). In the second stage, sampled cities were selected in each region randomly: Zhenjiang in the east; Chengdu in the west; Dongguan in the south; Yinchuan in the north. Shenmu was also selected as a sample city for its higher benefit of health care system than any other city in China. In the third stage, 1250 respondents were identified in these five cities. In each city, more than 200 respondents were randomly selected (231 in Zhenjiang, 201 in Dongguan, 297 in Chengdu, 253 in Shenmu and 216 in Yinchuan). All respondents were invited to finish the questionnaire and participate in the face-to-face interview. We collected the data from July 18, 2012 to August 20, 2012. Of the 1250 respondents surveyed, we excluded individuals who did not finish the questionnaire completely. This resulted in a total of 1198 respondents (617 males and 581 females; 449 urban workers, 336 urban residents and 413 rural residents) in our sample after excluding 52 respondents with missing values, yielding a response rate of 95.8%.

The same sampling method had been used in many similar surveys, such as Chinese provincial regional scale disease epidemiology survey conducted by Chen et al. targeted the self-rated multistage sampling design and The Statistical Methods and Applications of Two-stage Sampling Rotation Investigation conducted by Fu et al. also used this kind of method. Analyses of previous surveys suggest that this sampling method is adequate to generate a representative sample and its validity have been confirmed. Descriptive characteristics of respondents are reported in Table 1. In all, the survey was designed to encompass respondents from each of the three health insurance groups (rural, urban working and urban non-working).

**Table 1:** Descriptive characteristics of survey respondents

Characteristic		Proportion (%)
Cities	Zhenjiang	19.3
	Dongguan	16.8
	Chengdu	24.8
	Shenmu	21.1
	Yinchuan	18.0
Age distribution	40 and Below	57.2
	41–60	29.4
	61 and Above	12.2
	Not Available	1.2
Gender proportion	Male	51.5
Groups	Urban Workers	37.5
	Urban Residents	28.0
	Rural Residents	34.5
Occupational distribution	Civil Servants and Staff of Public Institutions	20.4
	Staff of Foreign-Funded and Private Enterprises	14.5
	Staff without Fixed Employment	59.3
	Retirees	5.8
Educational background	Primary School and Below	15.8
	Middle School	46.4
	Junior College and Above	37.8
Household registration types	Local	62.4
	Non-Local	14.4
	Not Available	23.2
One-year hospitalization rate	Yes	10.2
	No	87.1
	Not Available	2.7
Take Exercise	Frequently	27.8
	Sometimes	52.6
	Never	19.2
	Not Available	0.4
Monthly health condition	Very Good	16.4
	Good	36.6
	Average	38.8
	Poor	6.1
	Very Poor	0.8
	Not Available	1.3
Incidence of chronic disease	Yes	14.9
	No	85.1
Two-week morbidity rate	Yes	8.5
	No	88.5
	Not Available	3.0

### Variables and survey scale

In this paper, the dependent variable, ‘perceived benefit’ of health care, is defined as the weighted sum of the degree of satisfaction with four components of the health system: basic public health, health insurance, health insurance procedures and health services provision. The four aspects have their respective connotation. Basic public health includes resident health record, health education, vaccination, infectious diseases and health emergence, children health care, women health care, elderly health care, chronic diseases management, major psychosis management and health supervision and coordination service; health insurance includes insurance range, type of coverage, level of coverage reimbursement and satisfaction insurance procedures; health insurance procedures includes procedures, information, reimbursement, off-site review procedures and transfer & reviewed procedures; health services provision includes queue up to register, waiting times, medical procedures, doctor attitude, doctors level, environmental facilities, hospital charges and types of drugs. Based on expert opinion, the weights applied to each component are 0.3, 0.25, 0.15 and 0.3, respectively. As health insurance procedures were considered to be uniformly applied across respondents, this component is excluded from the analysis of variations in equality. Expert opinion is again used in a similar manner to that for the weighting of satisfaction with the components of equality. The respondents assessed their degree of satisfaction on a 5-point Likert scale, with a score of 1 meaning the lowest degree of satisfaction, while a score of 5 meaning the highest degree of satisfaction, the details can be seen in Table 2.

**Table 2:** Benefit with China Health Care

Health Care	‾S (s.d)	‾S_R_ (s.d)	‾S_U_ (s.d)
Basic Public Health	3.00 (0.779)	3.00 (0.690)	2.88 (0.827)
Resident health record	3.04 (0.894)	3.06 (0.801)	2.81 (0.909)
Health education	3.11 (0.869)	3.16 (0.819)	2.93 (0.939)
Vaccination	3.24 (0.844)	3.28 (0.811)	3.13 (0.935)
Infectious diseases and health emergence	3.06 (0.878)	3.12 (0.894)	2.94 (0.965)
Children healthcare	3.10 (0.906)	3.17 (0.904)	2.97 (0.941)
Women health care	3.07 (0.893)	3.13 (0.888)	2.92 (0.882)
Elderly health care	2.94 (0.964)	3.01 (0.919)	2.77 (1.042)
Chronic diseases management	2.89 (0.866)	2.98 (0.815)	2.76 (0.905)
Major psychosis management	2.90 (0.878)	2.96 (0.800)	2.83 (0.923)
Health supervision and coordination service	2.82 (0.954)	2.88 (0.891)	2.66 (0.928)
Health Insurance	2.97 (0.841)	2.96 (0.758)	2.70 (0.849)
Insurance range	3.11 (0.890)	3.06 (0.855)	2.87 (0.910)
Type of coverage	3.07 (0.866)	3.04 (0.838)	2.86 (0.893)
Level of coverage reimbursement	2.90 (0.929)	2.89 (0.859)	2.65 (0.925)
Satisfaction insurance procedures	2.89 (0.909)	2.93 (0.816)	2.62 (0.924)
Health Insurance Procedures	3.00 (0.811)	2.99 (0.729)	2.87 (0.795)
Procedures	3.35 (0.850)	3.33 (0.772)	3.29 (0.848)
Information	3.11 (0.827)	3.12 (0.792)	2.96 (0.831)
Reimbursement	2.89 (0.932)	2.88 (0.889)	2.76 (0.861)
Off-site review procedures	2.86 (0.851)	2.84 (0.787)	2.74 (0.745)
Transfer & reviewed procedures	2.89 (0.872)	2.90 (0.783)	2.84 (0.838)
Health Service	2.89 (0.793)	2.91 (0.734)	2.81 (0.803)
Queue up to register	2.95 (0.936)	2.9 (0.883)	2.84 (0.892)
Waiting times	2.91 (0.932)	2.89 (0.938)	2.83 (0.878)
Medical procedures	2.91 (0.905)	2.93 (0.872)	2.83 (0.899)
Doctor attitude	2.94 (0.969)	3.01 (0.954)	2.94 (0.974)
Doctors level	3.07 (0.901)	3.08 (0.899)	2.98 (0.915)
Environmental facilities	3.11 (0.858)	3.16 (0.826)	3.02 (0.958)
Hospital charges	2.58 (0.986)	2.58 (0.943)	2.34 (0.952)
Types of drugs	2.96 (0.853)	2.91 (0.820)	2.86 (0.891)

Strongly unsatisfied = 1, Less satisfied = 2, neutral =3, Very satisfied = 4, Strongly satisfied = 5

Similarly, ‘perceived equality’ within health system is measured through respondents’ perception of the fairness compared with the benefits that their counterparts achieved to the same system associated with each of three health system components: basic public health; health insurance; and the provision of health services, with each component weighted equally at 0.33, based again on expert opinion. As health insurance procedures are considered to be uniformly applied across respondents, this component is excluded from the analysis of variations in equality. Again, each of the three components are scored on a scale from 1 to 5, where a score of 5 meant a perception of complete equality (or fairness); 3 meant a neutral perception to equality; and 1, a situation of complete inequality (or unfairness). Respondents who obtained the higher scores tend to evaluate more equality/fairness on the health care. See Table 3.

**Table 3:** Equality with China Health Care

Health Care	‾D (s.d)	‾D_R_ (s.d)	‾D_U_ (s.d)
Basic Public Health	2.77 (0.835)	2.80 (0.755)	2.58 (0.791)
Resident health record	2.90 (0.973)	2.91 (0.927)	2.66 (0.906)
Health education	2.89 (0.931)	2.96 (0.867)	2.68 (0.924)
Vaccination	3.07 (0.945)	3.06 (0.893)	2.86 (0.996)
Infectious diseases and health emergence	2.90 (0.932)	2.90 (0.882)	2.74 (0.952)
Children health care	2.90 (0.975)	2.91 (0.930)	2.74 (0.982)
Women health care	2.87 (0.949)	2.85 (0.885)	2.71 (0.981)
Elderly health care	2.76 (0.975)	2.76 (0.907)	2.58 (1.040)
Chronic diseases management	2.77 (0.919)	2.79 (0.862)	2.62 (0.883)
Major psychosis management	2.78 (0.927)	2.81 (0.885)	2.61 (0.912)
Health supervision and coordination service	2.70 (0.937)	2.72 (0.890)	2.52 (0.864)
Health Insurance	2.79 (0.871)	2.73 (0.763)	2.51 (0.826)
Insurance range	2.95 (0.960)	2.89 (0.879)	2.62 (0.916)
Type of coverage	2.93 (0.926)	2.89 (0.873)	2.64 (0.917)
Level of coverage reimbursement	2.78 (0.957)	2.72 (0.866)	2.50 (0.910)
Satisfaction insurance procedures	2.75 (0.940)	2.73 (0.835)	2.47 (0.905)
Health Service	2.65 (0.869)	2.64 (0.782)	2.41 (0.827)
Hospital beds	2.82 (0.964)	2.83 (0.944)	2.60 (0.910)
Health personnel	2.72 (0.939)	2.75 (0.889)	2.45 (0.904)
Medical equipment	2.58 (0.997)	2.59 (0.923)	2.29 (0.925)
Medical technology	2.55 (0.961)	2.56 (0.892)	2.27 (0.857)
Pharmaceutical	2.72 (0.924)	2.66 (0.854)	2.51 (0.923)

Complete Inequality = 1, Some Inequality = 2, Neutral = 3, Some Equality = 4, Complete Equality = 5

### Analysis

Descriptive statistics are used to test the statistical differences in scores of benefit and equality within the health care system among respondents. Stratified analyses are conducted for the independent variables to determine whether the factors had association between rural/urban residence and health services utilization differed across strata (including benefit and equity). Specifically, we assign value 0 to the dependent variables, benefit and equality, if the score is below 3, which means dissatisfaction and inequality, respectively; we assign value 1 to the dependent variables if the score is 3 or above 3, meaning satisfaction or equality. Finally, based on the variate distribution of the dichotomous measures of perceived equality and benefit, respondents are classified into four groups: “high equality and high benefit”; “low equality and low benefit”; “high equality and low benefit”; and finally, “low equality and high benefit”.

Statistical models are employed to identify those variables that accounted for differences in perceived equality and benefit between urban and rural respondents. Determinants are analyzed by using logistic regression methods, and the following statistical analyses are calculated by using SPSS (version 20.0). Correlation Analysis has been conducted to ensure the regression model could be employed. Spearman rank correlation analysis is adopted on dichotomous variables to assess the correlations between alternative independent variables and the two dependent variables: perceived benefit and perceived equality. Then ANOVA is performed on variables which were found to be significantly correlated with perceived equality and perceived benefit for health care to measure benefit and equality scores according to different factors. The independent variables are based on significance in the correlation analysis and backwards step-wise regression is used to settle on a preferred model. The results are reported in Table 4. An array of alternative independent variables is reviewed and the variables that are significantly correlated with satisfaction and equality are selected for use in the analysis of variance.

**Table 4:** Results of variance analysis on survey of national health care

		Benefit and equality score	Benefit score	Equality score
Type of Health Insurance	F	5.088	6.176	2.160
	Sig.	<0.01	<0.01	>0.05
Health Change	F	19.302	11.174	18.058
	Sig.	<0.01	<0.01	<0.01
Fitness Condition	F	4.013	3.793	4.844
	Sig.	<0.05	<0.05	<0.01
Monthly Health Condition	F	10.184	5.703	4.761
	Sig.	<0.01	<0.01	<0.01
Cities	F	18.050	15.605	22.522
	Sig.	<0.01	<0.01	<0.01
Occupational Distribution	F	6.614	5.286	6.378
	Sig.	<0.01	<0.01	<0.01
Marital Status	F	5.447	2.662	5.545
	sig.	<0.01	>0.05	<0.01
Education	F	7.523	3.290	10.085
	Sig.	<0.01	<0.05	<0.01

## Results on perceived equality and Benefit from National Health Systems

### Overall results

Descriptive characteristics of respondents are reported in Table 1. Respondents were evenly distributed across the five cities wherein 51.5% are male and 42.8% of respondents are forty years old or more.

Table 2 shows the scores of respondents’ evaluation on the benefit of Health Care System in China. In this table, we list several waves of four aspects of Health Care System in China to make the respondents evaluate the degree of health system they obtained. From this result, we can see that respondents can achieve more benefit from basic public health and health insurance procedures than that from health insurance and health service. Table 3 shows the scores of respondents’ evaluation on the equality of Health Care System in China. In this table, we list several waves of three aspects of Health Care System in China to make respondents evaluate whether they had achieved equal benefit from this system compared with their counterparts. From this result, we can see that respondents thought they have achieved more equal benefit from health insurance, almost the same as basic public health, compared with that from health service.

On aggregate, perceptions regarding equality and benefit from China’s national health care system are divided into four groups with the distribution across these groups. Almost a third of respondents, specifically 30.4%, report high levels of benefit and equality within the Chinese health care system. At the other end of the spectrum, 43.0% of respondents report low levels of benefit and high levels of inequality within the health care system. There are two other classifications between these two extremes: 4.6% of respondents perceived high levels of equality, but low perceived benefits from the health care system; and 22.0% of respondents report high levels of benefit but high levels of inequality.

### Geographic results

The geographic results are shown in Table 5. In the low equality/low benefit group, respondents from Dongguan and Chengdu accounted for 59.90% and 58.17% of all respondents, respectively. In the high equality/low benefit group, most respondents are from Zhenjiang, Dongguan, Shenmu and Yinchuan, and only 2.4% of respondents from Chengdu. In the low equality/high benefit group, respondents from Yinchuan and Zhenjiang represented 37.41% and 37.30% of respondents, respectively. Finally, in the high equality / high benefit group, respondents from Shenmu represent 47.95% of all respondents. This shows that workers and residents in Shenmu enjoyed a higher level of benefit and equality of health care system than those in other places, and the situation of health care system in Chengdu was the most disappointing.

**Table 5:** The table of comprehensive results on equality and benefit

		Low Equality and low benefit	Low equality and high benefit	High equality and low benefit	High equality and high benefit
Regions	Zhenjiang	32.54%	37.30%	5.56%	24.60%
	Dongguan	59.90%	7.61%	5.58%	26.90%
	Chengdu	58.17%	22.60%	2.40%	16.83%
	Shenmu	30.74%	15.98%	5.33%	47.95%
	Yinchuan	28.57%	37.41%	4.08%	29.93%
Population Groups	Urban Workers	41.19%	20.75%	4.40%	33.65%
	Urban Residents	41.47%	22.87%	6.98%	28.68%
	Rural Residents	45.95%	22.54%	2.89%	28.61%
Age	Under 40	44.84%	18.20%	4.69%	32.27%
	40–60	38.91%	26.55%	4.00%	30.55%
	Over 60	42.31%	31.73%	5.77%	20.19%
Sex	Male Female	38.48% 48.46%	22.04% 21.99%	4.61% 4.49%	34.87% 25.06%

### Results based on population characteristics

In the low equality/low benefit group, rural residents account for higher proportion (45.95%) than urban residents (41.47%) and urban workers (41.19%) (in Table 5). In the high equality/low benefit group, rural residents account for the lowest proportion of only 2.89%, while urban residents who hold this evaluation account for the highest proportion, of 6.98%. For the low equality/high benefit group, the respondent populations are not significantly different. However, among respondents in the high equality/high benefit group, urban workers are over-represented as they perceived to have more access to quality care than other respondents. In general, the difference of evaluation on the benefit and equality of health care system among urban workers, urban residents and rural residents is not so remarkable that may be ignored.

From a demographic perspective, respondents ‘under 40’, middle-aged, and ‘over 60’ represent 44.84%, 42.31% and 38.91%, respectively, in the low equality/low benefit group (Table 5). In the case of the high equality/high benefit group, older respondents represent 20.19% of respondents of that category, fewer than ‘under 40’ and ‘40–60’ year-old respondents, at 32.27% and 30.55%, respectively. This shows that the elderly people made lower evaluations on the benefit and equality of health care system. It seems that old people were victims of the health care system.

## Factors that influenced variations in perceived equality and benefit

Table 6 reports binary logistic regression results for satisfaction and equality with the health system, in terms of satisfied/dissatisfied and equality/inequality. By comparing results of the scores of benefit and equality, it is found that the type of health insurance, education, occupational distribution, region and changes in health status over the course of one year affected both satisfaction and equality. Marital status and fitness only affect equality. Personal health status only influence satisfaction, but not equality.

**Table 6:** Results of variables logistic regression on benefit and equality of national health care

	Benefit (0 = dissatisfied 1 = Satisfied)	Equality (0 = Inequality 1 = equality)
	B (S.E.)	B (S.E.)
Type of health insurance (Reference Category: Others)		
Basic health insurance of Urban Employees	2.021** (0.523)	1.310** (0.439)
Basic health insurance of Urban and Rural Residents	2.211** (0.522)	1.188** (0.432)
Free Medical Service	1.999** (0.707)	1.057 (0.595)
Education (Reference Category: College and Above)		
Junior middle school and below	−.524** (0.203)	−.354 (0.200)
High School or Technical Secondary School	.043 (0.208)	.214 (0.193)
Marital Status(Reference Category: Unmarried)		
Unmarried		.888* (0.404)
Married		.447 (0.370)
Occupational Distribution(Reference Category: Others)		
Civil Servants and State-owned Enterprises and Institutions	.775** (0.241)	.342 (0.221)
Foreign and Private Enterprises	−.184 (0.264)	−.347 (0.251)
Workers without Fixed Employment	.295 (0.219)	.569** (0.211)
Retirees	.417 (0.329)	.244 (0.331)
Cities(Reference Category: Yinchuan)		
Zhenjiang	.007 (0.279)	−.022 (0.234)
Dongguan	−1.189** (0.280)	−.246 (0.248)
Chengdu	−1.129** (0.257)	−1.364 (0.241)
Shenmu	−.192 (0.251)	.609** (0.222)
Monthly Health Condition (Reference Category: Very Good)		
Very Poor	1.585* (0.783)	1.685 (0.873)
Poor	1.610* (0.768)	1.558 (0.861)
Ordinary	.985 (0.762)	1.256 (0.858)
Good	1.139 (0.802)	1.718 (0.896)
Fitness Condition (Reference Category: Never)		
Regularly		.324 (0.237)
Occasionally		.451* (0.205)
Health changes in nearly one year (Reference Category: Uncertain)		
Unchanged	.584** (0.218)	.455* (0.210)
Better	.827** (0.238)	.919** (0.229)
Poorer	.354 (0.260)	−.194 (0.253)
Constant	−3.653** (1.04)	−3.044** (0.655)
Reference Category	Dissatisfied	Inequality
Selected Cases	863	1109
-2LL	1051.049	1224.590
Preudo R2 (Cox & Snell)	0.152	0.158
Predicted percentage correct	66.3%	72.0%

(** means ‘*P* value < =0.01’, * means ‘*P* value < =0.05’)

Factors of health insurance have significant impacts on benefit and equality. Compared to the residents who participated in health care assistance or business health insurance (the reference group), residents who participated in urban basic medical insurance system for workers, urban medical insurance for residents (non-workers), and public-cost health security system (for civil servants) perform positive attitude toward satisfaction. In terms of education, compared with the reference group, respondents in the group of middle school and below have a lower probability to hold positive attitudes regarding satisfaction. The factor of occupation is an important element that affected two evaluations. Civil servants, workers, and staff of state-owned enterprises and institutions are more likely to be satisfied with the system compared with farmers, students and unemployed residents. Among all these cities, compared with respondents in Manchurian, respondents in Chengdu and Guangzhou are more likely to hold a negative attitude to satisfaction. A logistic regression analysis is conducted to demonstrate the degree that these factors affected the benefit and equality among different groups of populations. Concerning equality, the probability of holding a positive attitude in Sheen is 1.838 times higher than that of Manchurian, which indicates that Sheen may do better than Manchurian in the health care system. Concerning the change in health status, those who don’t report a change in their health status in the past year are more likely to report a positive attitude to the health system compared with the group whose health status is getting worse because they had felt the benefit of this system. Monthly health condition is a factor that only affected satisfaction. However, the analysis of regression results indicates that the impact of monthly health condition on satisfaction are not stable. Compared with people in good health, those with poor health reported higher satisfaction. Marital status and fitness condition are factors which only affected equality. In terms of marital status, unmarried residents are more likely to be satisfied with the equality degree compared with the married group. Compared with people who never took part in physical exercise, those who took exercise more than three times a week are more likely to hold a more positive attitude towards equality (1.382 times higher).

## Discussion

The results indicate that both urban and rural residents have worse evaluation on benefit and equality of health care system in China. Compared with previous research, this study is the only one with largest-scale survey on public health, which includes each geographical part in China and the largest sample size survey on public health satisfaction in China. Du and Wang [23] conducted a survey about public health services in the rural area leading to a similar conclusion that public health system had shortcomings with lower citizen satisfaction due to its socioeconomic insufficient development. Xu and Deng [24] revealed in his research on public health that people in rural were more satisfied with health services financed publicly than people in urban area. Zhu et al. [25] conducted a research about other area of public services with the factor analysis, but his research was lacking of superior methods. Ying et al. [26] and Li and Zhong [27] both got the result that public health services in rural area were different from urban but they didn’t conclude the leading factors of this consequence and their data were not representative. All the previous related survey all focus on a partial direction or orientation other than a comprehensive perspective. The data from this survey is very unique and special among all associated research. Moreover, the conclusion pertaining to the discrepancy of public health services between urban and rural is unprecedented based on the reliable and effective data. The differences are apparent for broad subjective measures of health care system (overall self-assessed survey). We also find that the disparities in health care treatment among different groups of populations have been declining over time. Unfortunately, this appears to reflect relatively more dramatic declines for those who didn’t have more opportunities to obtain higher education and be employed, especially in developing areas. Deteriorating evaluation on benefit and equality of health care system has occurred alongside declines in the treatment of basic health insurance. This trend is especially pronounced among urban residents. Soaring health care costs may be a major reason that contributes to this decline in evaluation of health care system.

Our results highlight that the importance of the type of health insurance, education, occupation and geographic regions, which are key determinants of perceived levels of equality and benefit derived from the Chinese national system of health care. As mentioned above, residents who achieved more treatment from the system have a high education and are employed so that can approve the current system more easily. Compared to their rural counterparts, urban workers and residents have easier and more access to benefit from the development of the society, thus display a more satisfied attitude towards this system than rural residents as a whole.

Several limitations should be noticed. First, the study is based on a sample of 1250 urban and rural residents from five cities of China. Although these cities sampled are nationally representative of the country, one should be cautious when to generalize it to the whole country. However, this research can pose the situation at the region-level to some extent. In future work, we hope to expand the scope of the survey and increase the number of respondents in order to make more confident generalizations for China. Second, not all survey respondents completed the survey in its entirety, resulting in missing values of some variables for some respondents. In this situation, based on multiple imputation methods like descriptive statistics, we decide to exclude respondents with missing values. Overall, this has a relatively small impact on the analysis sample as it accounts for over 4.2% of the survey respondents. However, we believe that the missing values cannot change our results.

## Conclusions

The evaluation of satisfaction with the health care is very important, helping us to improve health services and promote individual health psychologically physiologically. From our findings, we find several factors including the type of health insurance, education, occupation, and geographic region, which explained the variation in perceived benefits from respondents and which affected equality of the Chinese public health system.

The findings illustrates wide variation in perceptions of equality and benefit between local and rural residents and across population characteristics, leading to a perceived lack of fairness in benefits and accessibility to urban dwellers.

Based on our research, we find that the type of health insurance, educational qualifications, and occupational distribution play important roles on perceived equality and benefit for health care among urban and rural residents. This study is conducive public health policy implementation. Although China has achieved remarkable economic successes in the past decade, the evidence on health care status and access appears to be far less impressive. Rising health care costs, a deteriorating environment, and inadequate health insurance coverage may play an important role in these patterns. More research is needed to identify the critical reasons for different degrees on benefit and equality among different group of populations, and the declining satisfaction and benefit in health care system over time are apparent in both urban and rural areas.
